# Supplementary material for: Barcoded Rabies In Situ Connectomics for high-throughput reconstruction of neural circuits
Source: Nat Commun. 2026 Sep 2;17:10463. doi: 10.1038/s41467-026-77105-y (PMC13635221; doi:10.1038/s41467-026-77105-y)
Supplement: Supplementary file 1 [file 41467_2026_77105_MOESM1_ESM.pdf]

Supplementary Information for: Barcoded Rabies In Situ Connectomics for high-throughput reconstruction of neural circuits

Alexander Becalick[1,*], **Antonin Blot**[1,*], **Molly Strom**[2], **and Petr Znamenskiy**[1,✉]

[1]Specification and Function of Neural Circuits Laboratory, The Francis Crick Institute, United Kingdom
[2]Viral Vector Service, The Francis Crick Institute, United Kingdom
[*]These authors contributed equally

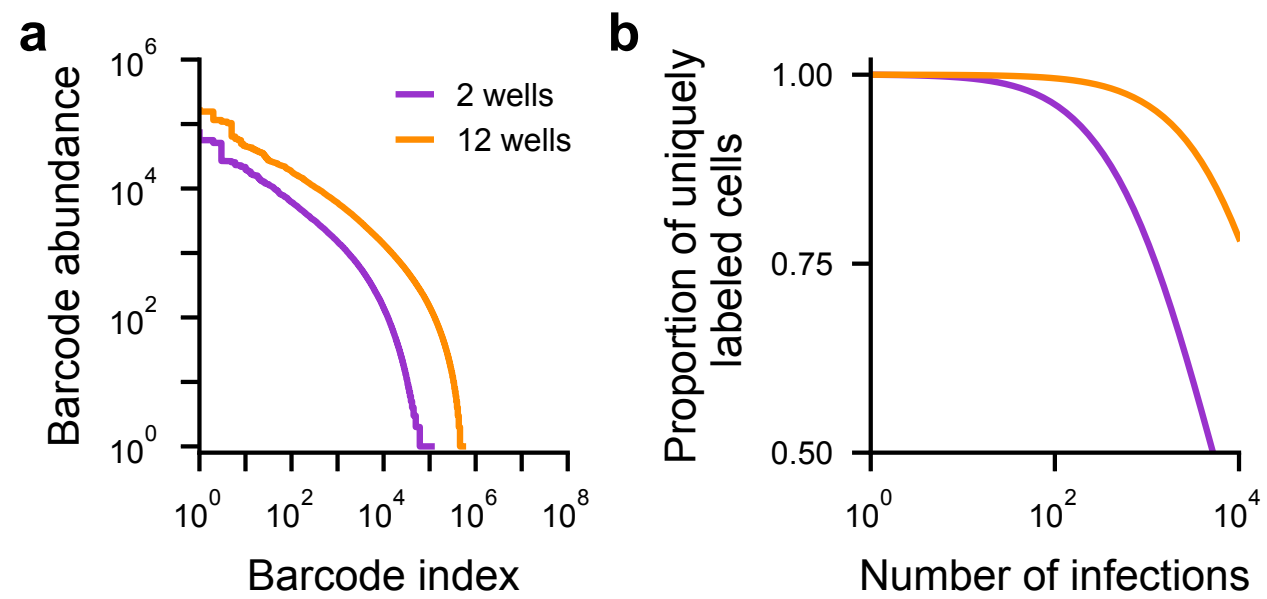

Supplementary Figure 1. Library complexity and the number of neurons that can be uniquely labeled with a library depend on the library production scale. a, Barcode abundance distribution of two viral libraries. Barcodes are sorted according to their abundance in the sequencing library, estimated using unique molecular identifiers (see Methods). **b**, Proportion of uniquely labeled starter neurons as a function of the number of independent infections.

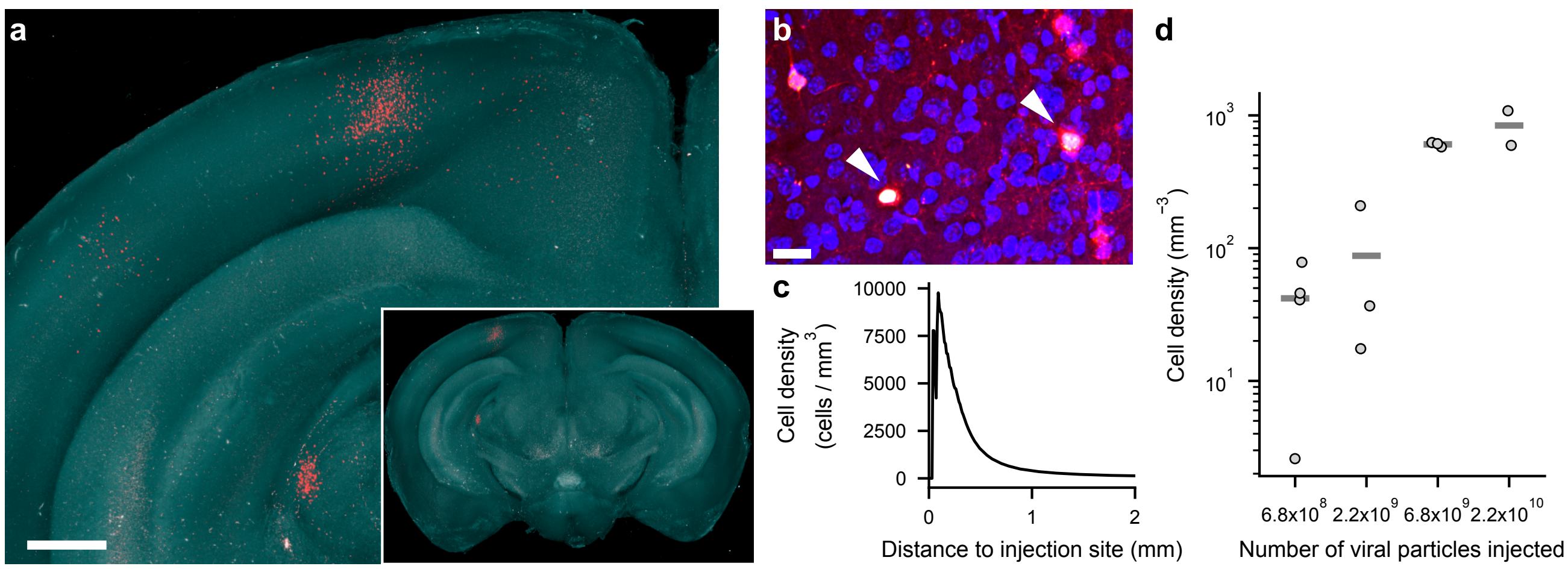

Supplementary Figure 2. Presynaptic cell distribution and starter cell density with intravenous Cre delivery. **a**, mCherry fluorescence in a section of mouse brain infected with the barcoded rabies virus library in the primary visual cortex with Cre delivered by intravenous injection, imaged using serial two-photon tomography. Maximum intensity projection over 1.2 mm. Scale bar – 1 mm. **b**, Identification of starter neurons based on nuclear mCherry fluorescence in a confocal microscopy image (maximum intensity projection spanning 9 µm, blue – DAPI, red and green – mCherry channel with different contrast limits, see Methods). Scale bar – 20 µm. 84.4 presynaptic neurons per starter (2025 presynaptic neurons for 24 starter cells) were detected, with 32.9 presynaptic cells per starter within 0.5 mm of the injection center. **c**, Density of rabies-infected neurons as a function of distance to the injection site. **d**, Density of tdTomato-positive cells in the visual cortex of Ai14(RCL-tdT)-D Cre reporter mice after tail vein injections of serial dilutions of AAV-PHP.eB-Cre. Bars represent mean density (N = 4 mice for 6.8 x 10^8 vg, N = 3 mice for 2.2 x 10^9, 6.8 x 10^9 vg, N = 2 for 2.2 x 10^{10} vg).

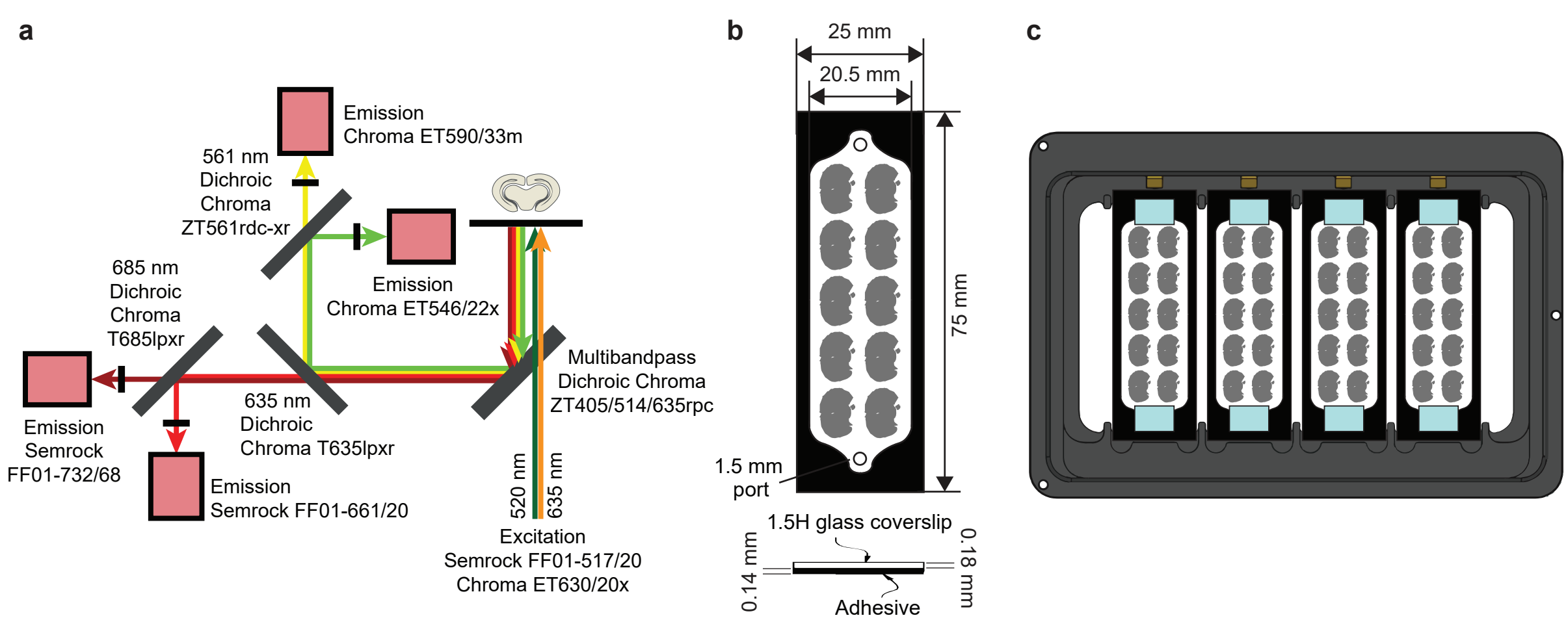

Supplementary Figure 3. Setup for high-throughput *in situ* sequencing. a, Diagram of the light path of the *in situ* sequencing microscope for imaging four channels simultaneously. **b,** Diagram of a microscope slide sized flowcell with a top-down view above and a side-on view below showing mouse coronal brain sections to scale. **c,** Diagram of the flowcells in the 4-slide microscope stage attachment used for the sequencing microscopes.

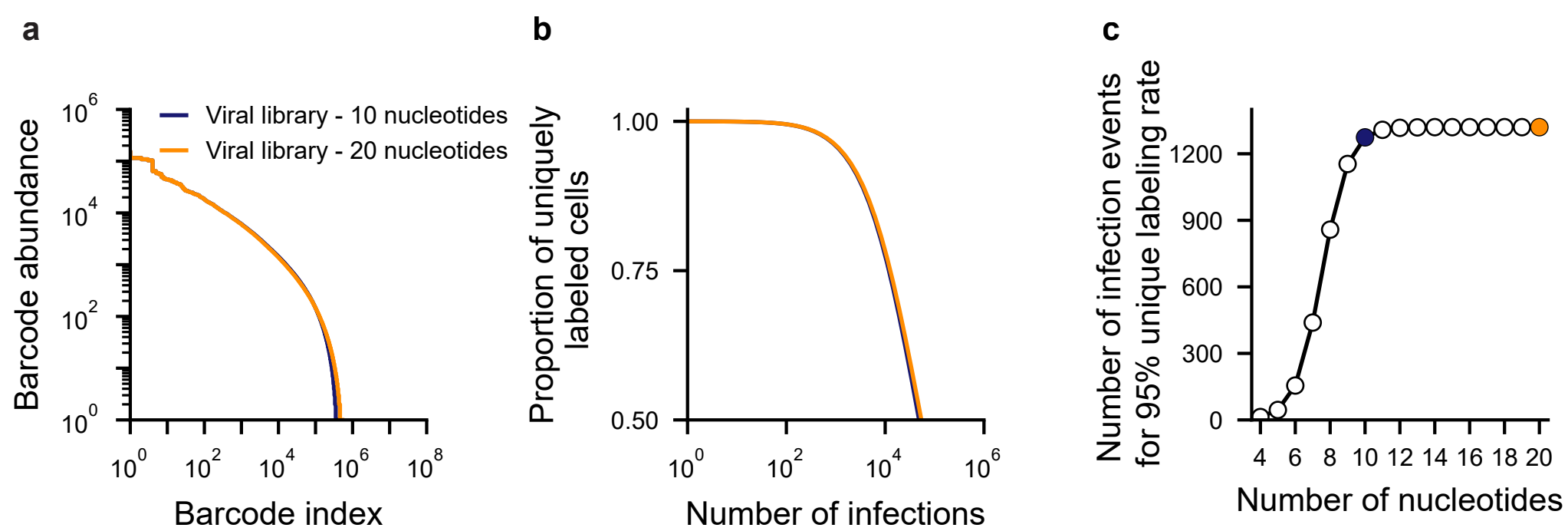

Supplementary Figure 4. Sequencing 10 nucleotides of the viral barcode is sufficient to uniquely identify infected cells. a, Barcode abundance distribution of plasmid and viral libraries as in Fig. 1d, quantified using the full barcode sequence (orange) and the first 10 nucleotides of the barcode (blue). **b,** Proportion of uniquely labeled starter neurons as a function of the number of independent infections, as in Fig. 1e. **c,** Maximum number of cells that can be labeled with at least 95% of uniquely labeled cells as a function of barcode length. Colored dots indicate the full library length (20 nucleotides, orange) and the length sequenced *in situ* (10 nucleotides, blue)

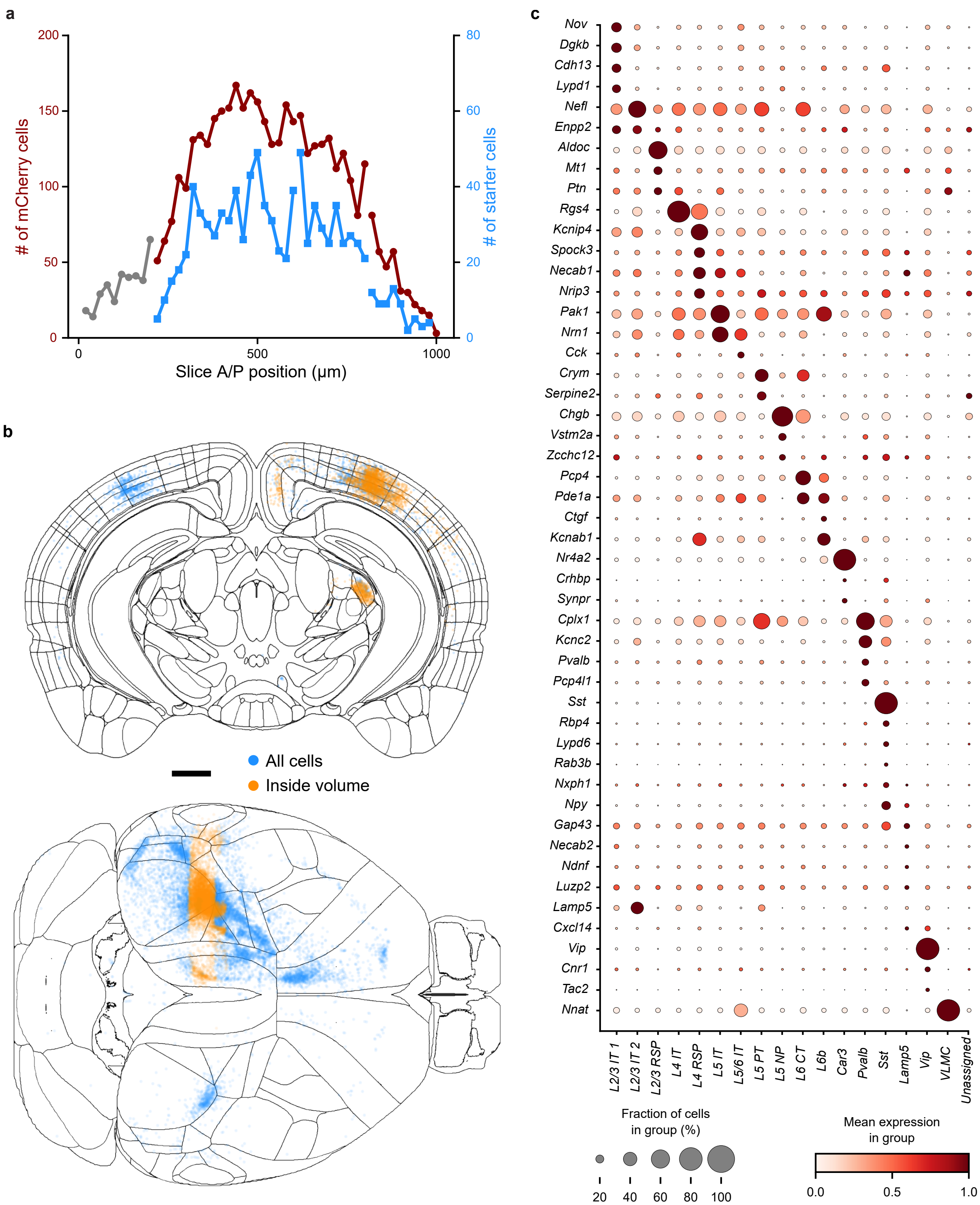

Supplementary Figure 5. Proportion of cells captured within the sequenced volume and gene expression across clusters. a, Number of mCherry cells (circles) detected in each section in sequenced sections (red) or non-sequenced sections (grey) as a function of the antero-posterior section position. The number of rabies-infected starter cells (blue square, right y-axis) found in each section follows the distribution of the total number of mCherry cells per section (infected and uninfected). Line breaks indicate tissue sections in different flowcells. **b,** Coronal (top) and dorsal (bottom) projections of bulk-labeled rabies cell locations. The extent of the *in situ* sequencing volume was centered on the bulk-labeled injection site, and cells were classified as being within the extent of the volume (orange, 8,157 cells) or outside of the volume (blue, 10,160 cells). Scale bar 1 mm **c,** Dotplot of mean gene expression across all cell type clusters for a selection of 49 marker genes from the total panel of 98 genes with cluster-specific expression. Mean normalized and log1p-transformed expression of each gene was rescaled to 1 across cell-type clusters.

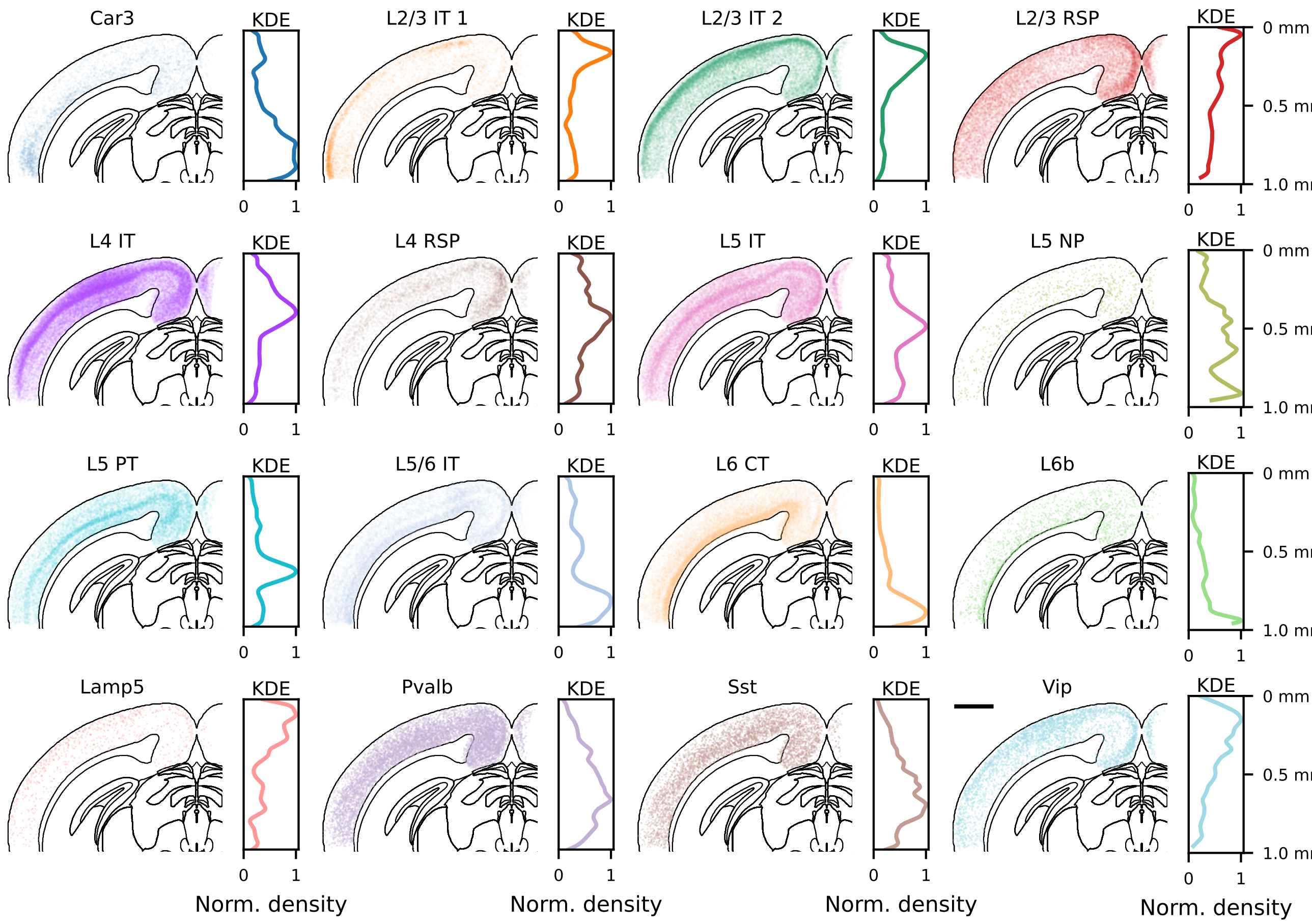

Supplementary Figure 6. Spatial location of identified cell-type clusters in cortex. Coronal projections of the spatial location of cells found in the cortex, grouped by assigned cell-type cluster. Scale bar – 1000 µm. Each scatter plot is accompanied by a kernel density estimate (KDE) of the distribution of cell depths normalized from the surface of the cortex (top) to the white matter boundary (bottom) of the flattened cortical coordinates for each cluster.

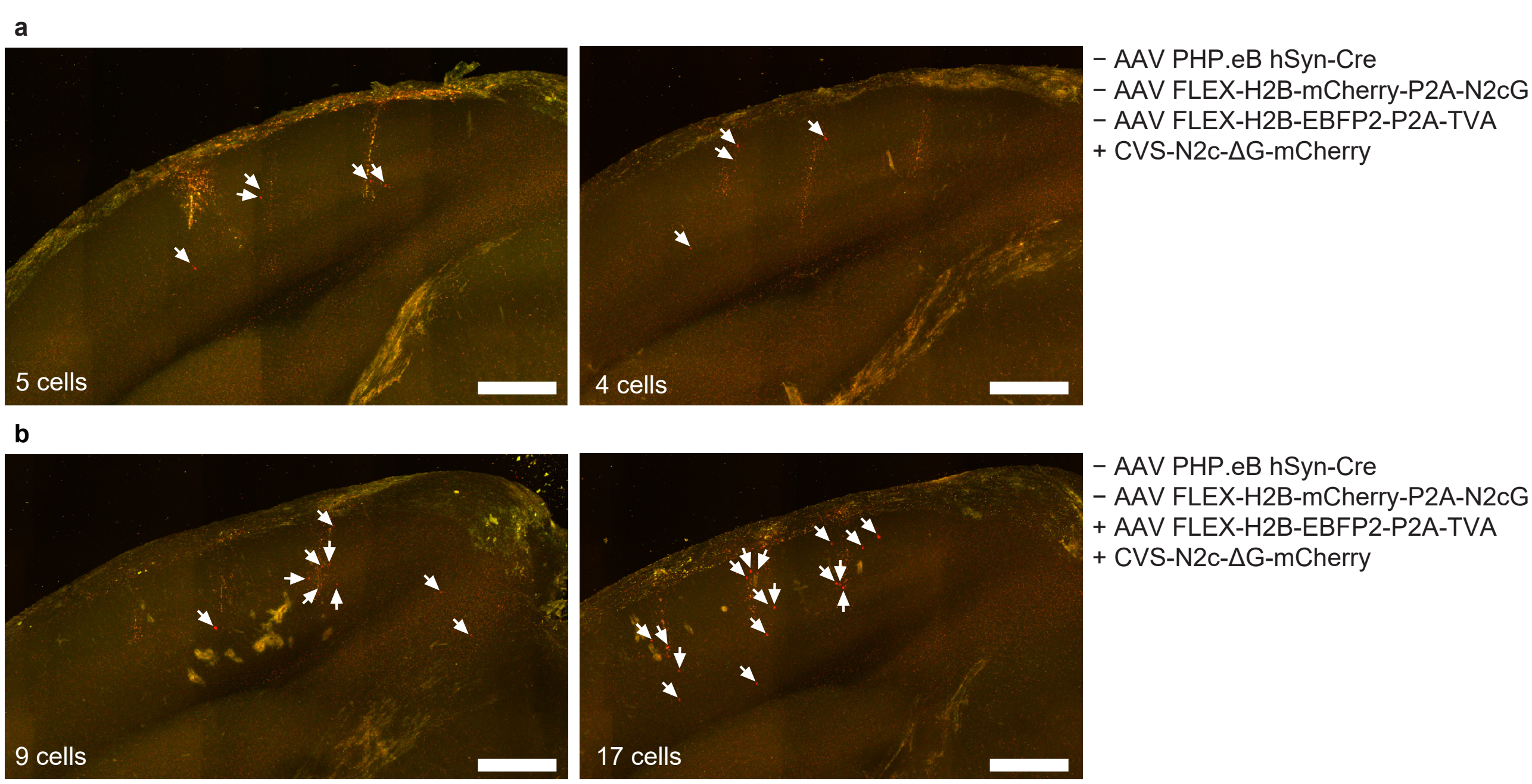

Supplementary Figure 7. Control experiments for direct infection by rabies viruses from glycoprotein-coated rabies contamination or AAV-TVA leak. a, Serial two-photon maximum coronal z-projections displaying 1 mm thickness of tissue centered on the injection sites of barcoded rabies in two brains. No TVA/glycoprotein/Cre virus injections were performed. **b,** Maximum z-projection through 1 mm of tissue from injection sites of both barcoded rabies and AAV-TVA in two brains. White arrows show the location of all detected rabies-infected cells. Scale bar – 500 µm.

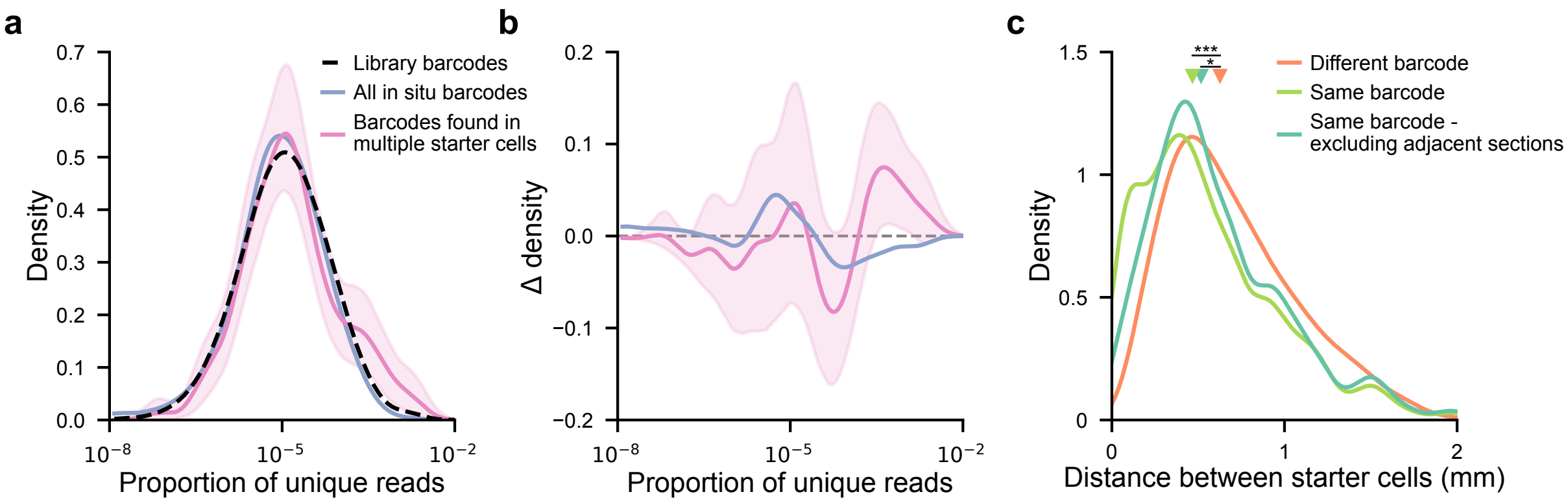

Supplementary Figure 8. Barcodes found in multiple starters are more abundant in the library and cluster spatially *in situ*. a, Kernel density estimates of the representation of barcodes as a proportion of unique reads in the viral library for all library barcodes (black, data as in Fig. 3b), all barcodes found in starter cells *in situ* (blue) or barcodes found in multiple starter cells *in situ* (pink). Shading indicates 95% confidence intervals computed by bootstrap resampling of barcodes found in multiple starter cells. **b,** Difference between the kernel density estimates for all barcodes or barcodes found in multiple starter cells and that of the library barcodes. Shading indicates 95% confidence intervals, computed by bootstrap resampling of barcodes found in multiple starter cells. **c,** The distance between starter cells containing the same barcode (light green, median 0.47 mm, 122 barcodes, 277 starter cells, 297 distances) was smaller than between these starter cells and starter cells with a different barcode (orange, median = 0.63 mm, 564 barcodes, 579 starter cells, 173,384 distances, p<0.001). This difference remained significant after excluding starter cells with the same barcodes found in adjacent sections (turquoise, median = 0.52 mm, 94 barcodes, 135 starter cells, 239 distances, p=0.028) and is therefore not due to duplicate detection of starter cells. Shading indicates 95% confidence interval, triangles indicate medians.

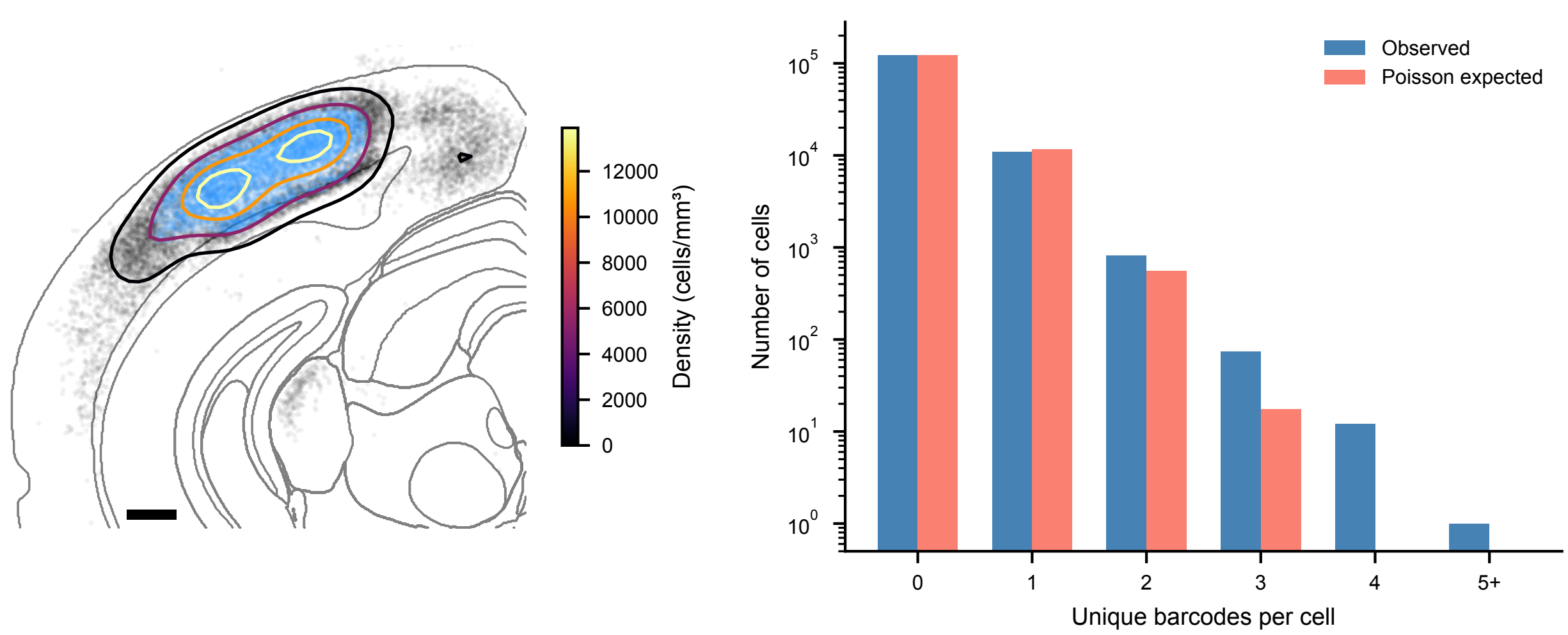

Supplementary Figure 9. Barcode count distribution does not show sign of superinfection exclusion. a, Density (contours) and distribution (black dots) of barcoded cells. Area outlines correspond to the coronal plane at the center of the sequencing volume. Scale bar – 1 mm. **b**, The number of cells with two or more barcodes (blue bars) for all barcoded cells in the area with 50% of the peak cell density (blue dots in **a**) was higher than that expected from a Poisson distribution (red bars) with the same mean (observed 903, expected 590.50, surplus = 312.50, p = 0.0004, ratio = 1.53, 95% percentile bootstrap CI = [1.45, 1.61]). The number of neurons with 0 barcodes for the observed distribution was estimated assuming neuron density of 150,000 neurons/mm³.

Supplementary Table 1. Library cloning and sequencing primers

Primer	Description	Sequence
Supercutter_F2	HDVagrz supercutter F primer	AGCCAGGCGGGGATCCGGCTGC
Supercutter_R	HDVagrz supercutter R primer	CTCCCTTAGCCATCCGAGTGGACG
Barcode_F	20 bp inverse PCR barcode primer F	GATCTGCGGCCGACTGCNNNNNNNNNNNNNNNNNNNNGGCGC CTAGCCGGTCATCC
Barcode_R	20 bp inverse PCR barcode primer R	ATGCAGCGGCCGACCGGCCGGGGTGGACTCAGGGC
Bipartite_barcode_F	20 bp inverse PCR bipartite barcode primer F	GATCTGCGGCCGACTGCNNNNNNNNNGGCGCCTAGCCGGTCATCC
Bipartite_barcode_R	20 bp inverse PCR bipartite barcode primer R	ATGCAGCGGCCGACCGGNNNNNNNNNCCGGGGTGGACTCAGGGC
UMI_P5_Truseq	UMI barcode library primer	CTACACGACGCTCTTCCGATCTNNNNNNNNNNNNGAAATGCCC TGAGTCCACCC
P7_universal reverse_primer	P7 library primer	GTGACTGGAGTTCAGACGTGTGCTCTTCCGATCACGACCATGTC TGATCAACTG
IDT_i5_X	i5 UDI index library primer	AATGATACGGCGACCACCGAGATCTACACNNNNNNNNACACTCT TTCCCTACACGACGCTCTTCCGATC
IDT_i7_X	i7 UDI index library primer	CAAGCAGAAGACGGCATACGAGATNNNNNNNNGTGACTGGAGT TCAGACGTGTG

Supplementary Table 2. *In situ* sequencing oligonucleotides

Primer	Description	Sequence
OAB0973	Padlock for the rabies barcode	/5Phos/GGCGCCTAGCCGGTCATCCTTCCTCTATGATTACTGAC TGCGTCTATTTAGTGGAGCATTGCTATCATTGCTATCTTCTTTC CACCCCGGCCGACTGC
RTAB0067	RT primer for the rabies barcode	/5AmMC12/G+AG+GA+GG+TT+AT+CC+TCGGGACTGGAATCGTC
PAB0010	RCA primer for Gad1	GGCATCTAACGGCTCTTAGTCAACTCTAC
PAB0012	RCA primer for Slc17a7	GCAATAAGTGCCGCGTGCTCGACG
PAB0013	RCA primer for Vip	GACGATCTGTCGACAACTATTAGTACAGCG
PAB0022	RCA primer for the V1 genes	CGACAGGTTCAGAGTTCTACAGTCCGACGAT
PAB0033	RCA primer for Sst	GAAGCGGTCAGCAACTGGATCGTG
PAB0035	RCA primer for the rabies barcode	GGCCGGGGTGGAAAGAAGATAGCAATG
PAB0023	Hybridization probe for the rabies barcode	/5ATTO532N/CCTCTATGATTACTGACTGCGTCTA
PAB0025	Hybridization probe for Slc17a7	/5ATTO565N/AGTCAGCGTCGAGCACGCGGCACTTATTGCA
PAB0026	Hybridization probe for Sst	/5ATTO532N/ATTGCACGATCCAGTTGCTGACCGCTTCAG
PAB0027	Hybridization probe for Vip	/5ATTO633N/TCGCTGTACTAATAGTTGTCGACAGATCGTCA
PAB0028	Hybridization probe for Gad1	/5ATTO700N/TGAGTAGAGTTGACTAAGAGCCGTTAGATGCC
PAB0029	Hybridization probe for the rabies barcode	/5ATTO565N/CCTCTATGATTACTGACTGCGTCTA
PAB0030	Hybridization probe for V1 genes	/5ATTO565N/ATCGTCGGACTGTAGAACTCTGAACCTGTCG
PAB0048	Hybridization probe for the rabies genes	/5ATTO633N/AGGATAGCATGACTGACGCTGATC
PAB0032	Sequencing primer for the V1 genes	A+TCG+TCG+GAC+TGT+AGA+ACT+CTGAACCTGTCG
PAB0034	Sequencing primer for the rabies barcode	TTGCTATCTTCTTTCCACCCCGGCCGACTGC
