# Supplementary material for: Barcoded Rabies In Situ Connectomics for high-throughput reconstruction of neural circuits
Source: Nat Commun. 2026 Sep 2;17:10463. doi: 10.1038/s41467-026-77105-y (PMC13635221; doi:10.1038/s41467-026-77105-y)
Supplement: Supplementary file 2 [file 41467_2026_77105_MOESM2_ESM.pdf]

Description of Additional Supplementary Files

Supplementary Data 1

Tables of sequences for the padlock probes, reverse transcription primers, readout primer and oligonucleotides used for in situ sequencing, as well as sequences for the plasmids used for viral production. The tables for in situ oligonucleotides are organised as follows: Name, Sequence, Synthesis scale, Purification, Gene (target). For plasmids, sequences are listed for all new plasmids generated in this study, while for existing plasmids the origin of the plasmid is listed.
