# Supplementary material for: Barcoded Rabies In Situ Connectomics for high-throughput reconstruction of neural circuits
Source: Nat Commun. 2026 Sep 2;17:10463. doi: 10.1038/s41467-026-77105-y (PMC13635221; doi:10.1038/s41467-026-77105-y)
Supplement: Supplementary file 3 [file 41467_2026_77105_MOESM4_ESM.pdf]

nature portfolio

Corresponding author(s): Petr Znamenskiy
Last updated by author(s): 14/08/2026

Reporting Summary

Nature Portfolio wishes to improve the reproducibility of the work that we publish. This form provides structure for consistency and transparency in reporting. For further information on Nature Portfolio policies, see our Editorial Policies and the Editorial Policy Checklist.

Statistics

For all statistical analyses, confirm that the following items are present in the figure legend, table legend, main text, or Methods section.

n/a	Confirmed	
☐	☒	The exact sample size (*n*) for each experimental group/condition, given as a discrete number and unit of measurement
☒	☐	A statement on whether measurements were taken from distinct samples or whether the same sample was measured repeatedly
☐	☒	The statistical test(s) used AND whether they are one- or two-sided<br>*Only common tests should be described solely by name; describe more complex techniques in the Methods section.*
☒	☐	A description of all covariates tested
☐	☒	A description of any assumptions or corrections, such as tests of normality and adjustment for multiple comparisons
☐	☒	A full description of the statistical parameters including central tendency (e.g. means) or other basic estimates (e.g. regression coefficient) AND variation (e.g. standard deviation) or associated estimates of uncertainty (e.g. confidence intervals)
☐	☒	For null hypothesis testing, the test statistic (e.g. *F*, *t*, *r*) with confidence intervals, effect sizes, degrees of freedom and *P* value noted<br>*Give P values as exact values whenever suitable.*
☒	☐	For Bayesian analysis, information on the choice of priors and Markov chain Monte Carlo settings
☐	☒	For hierarchical and complex designs, identification of the appropriate level for tests and full reporting of outcomes
☐	☒	Estimates of effect sizes (e.g. Cohen's *d*, Pearson's *r*), indicating how they were calculated

Our web collection on statistics for biologists contains articles on many of the points above.

Software and code

Policy information about availability of computer code

Data collection	In situ sequencing data was acquired using µManager (2.0.3, doi: 10.1002/0471142727.mb1420s92) and Pycromanager (v1.0.0, doi: 10.1038/s41592-021-01087-6). Two-photon serial sectioning data was acquired using StitchIt (10.5281/zenodo.3941901)
Data analysis	Original code required to reproduce the analyses has been deposited on Zenodo at https://doi.org/10.5281/zenodo.21499113. Code for processing raw in situ sequencing data is available at https://github.com/znamlab/iss-preprocess and https://github.com/znamlab/iss-analysis.

For manuscripts utilizing custom algorithms or software that are central to the research but not yet described in published literature, software must be made available to editors and reviewers. We strongly encourage code deposition in a community repository (e.g. GitHub). See the Nature Portfolio guidelines for submitting code & software for further information.

Data

Policy information about availability of data

All manuscripts must include a data availability statement. This statement should provide the following information, where applicable:

- Accession codes, unique identifiers, or web links for publicly available datasets
- A description of any restrictions on data availability
- For clinical datasets or third party data, please ensure that the statement adheres to our policy

The raw and processed library sequencing data have been deposited on the GEO database under accession code GSE302891. All other data required to reproduce the analyses, including preprocessed in situ sequencing data, have been deposited on Figshare at https://doi.org/10.25418/crick.29591696.
The raw in situ sequencing image datasets are not deposited in a public repository due to their prohibitive size (~150 TB). They are available for research purposes

from Petr Znamenskiy petr.znamenskiy@crick.ac.uk.

Research involving human participants, their data, or biological material

Policy information about studies with human participants or human data. See also policy information about sex, gender (identity/presentation), and sexual orientation and race, ethnicity and racism.

Reporting on sex and gender	N/A
Reporting on race, ethnicity, or other socially relevant groupings	N/A
Population characteristics	N/A
Recruitment	N/A
Ethics oversight	N/A

Note that full information on the approval of the study protocol must also be provided in the manuscript.

Field-specific reporting

Please select the one below that is the best fit for your research. If you are not sure, read the appropriate sections before making your selection.

[x] Life sciences [ ] Behavioural & social sciences [ ] Ecological, evolutionary & environmental sciences

For a reference copy of the document with all sections, see nature.com/documents/nr-reporting-summary-flat.pdf

Life sciences study design

All studies must disclose on these points even when the disclosure is negative.

Sample size	No sample size calculation was performed.<br><br>1h-k and Ext2a-c single bulk sample, 1l-n single sample per PHP/AAV1 condition, 3e single barcode vs rabies gene count sample, 3,4,5,6 all from single brain, Ext2d N = 4 mice for 6.8 x 10^8 vg/ml, N = 3 mice for 2.2 x 10^9, 6.8 x 10^9 vg/ml, N = 2 for 2.2 x 10^10 vg/ml, Ext5b 1 bulk brain, Ext7a-b controls 2 mice each
Data exclusions	No animals were excluded from analyses. Inclusion criteria for analysis of cells are described in the Methods.
Replication	The connectomic analysis from this study (Fig. 3,4,5,6) comes from a single sample and was not replicated. Quantification of starter cell density with AAV concentration (Extended data Fig. 2c) was replicated across n>3 animals per dilution. Control experiments for direct rabies infection (Extended data Fig. 7a,b) were replicated with n=2 animals per condition.
Randomization	Mice were randomly assigned to experimental groups for different injection conditions (Fig. 1l-n, Extended data Fig. 7a,b).
Blinding	Blinding was not performed for quantifying cell count/position for Fig. 1l-n. Cells were detected automatically, all data was processed with the same analysis pipeline. Blinding was not performed for Extended data Fig. 7a,b. Cells were counted manually.

Reporting for specific materials, systems and methods

We require information from authors about some types of materials, experimental systems and methods used in many studies. Here, indicate whether each material, system or method listed is relevant to your study. If you are not sure if a list item applies to your research, read the appropriate section before selecting a response.

Materials & experimental systems

n/a	Involved in the study	
x		Antibodies
	x	Eukaryotic cell lines
x		Palaeontology and archaeology
	x	Animals and other organisms
x		Clinical data
x		Dual use research of concern
x		Plants

Methods

n/a	Involved in the study	
x		ChIP-seq
x		Flow cytometry
x		MRI-based neuroimaging

Eukaryotic cell lines

Policy information about cell lines and Sex and Gender in Research

Cell line source(s)	HEK293T (ATCC cat. no. CRL-3216)<br>HEK293TG (in house stable cell line expressing rabies glycoprotein, derived from ATCC CRL-3216)<br>HEK293T-EnvA (doi: 10.1038/nprot.2013.094)<br>BHK-EnvA (doi: 10.1038/nprot.2013.094)
Authentication	Species identification and STR profiling was carried out on all cell lines
Mycoplasma contamination	All cell lines tested negative for mycoplasma contamination
Commonly misidentified lines<br>(See ICLAC register)	N/A

Animals and other research organisms

Policy information about studies involving animals; ARRIVE guidelines recommended for reporting animal research, and Sex and Gender in Research

Laboratory animals	Male and female C57Bl/6 mice aged between 12 and 15 weeks were used for barcoded rabies experiments. Ai14-tdTomato Cre-responder mice (B6;129S6-Gt(ROSA)26Sortm14(CAG-tdTomato)Hze/J, JAX \#007908) were used for AAV-PHP.eB-Cre titration experiments.
Wild animals	N/A
Reporting on sex	The connectomic findings in this study originate from a single male mouse. The sex of all animals selected for experiments was random.
Field-collected samples	N/A
Ethics oversight	All animal procedures were conducted in compliance with the guidance and regulations set forth by the UK Home Office according to the Animals Scientific Procedures Act 1986 (PPL PP4882546) and approved by the Animal Welfare Ethical Review Body at the Francis Crick Institute.

Note that full information on the approval of the study protocol must also be provided in the manuscript.

Plants

Seed stocks	N/A
Novel plant genotypes	N/A
Authentication	N/A
