# Supplementary material for: Barcoded Rabies In Situ Connectomics for high-throughput reconstruction of neural circuits
Source: Nat Commun. 2026 Sep 2;17:10463. doi: 10.1038/s41467-026-77105-y (PMC13635221; doi:10.1038/s41467-026-77105-y)
Supplement: Supplementary file 4 [file 41467_2026_77105_MOESM5_ESM.pdf]

nature portfolio

Peer Review File

Barcoded Rabies In Situ Connectomics for high-throughput reconstruction of neural circuits

Corresponding Author: Dr Petr Znamenskiy

This file contains all reviewer reports in order by version, followed by all author rebuttals in order by version.

Version 0:

Reviewer comments:

Reviewer #1

(Remarks to the Author)
The paper by Becalick et al describes the development of Barcoded Rabies In Situ Connectomics (BRISC), a method for high-throughput mapping of neural connections. The technique uses rabies viruses carrying random molecular barcodes to simultaneously trace the synaptic inputs of hundreds of individual neurons. The authors generated high-diversity barcoded rabies libraries capable of uniquely labeling over 1,000 neurons, controlled starter neuron density through systemic AAV delivery to minimize barcode transmission between starters, and developed an in situ sequencing approach to read out barcodes while preserving spatial information. Applied to mouse primary visual cortex, BRISC mapped inputs onto 385 starter neurons, identifying 7,814 putative synaptic connections. The results revealed layer-specific and cell-type-specific local connectivity patterns consistent with known V1 circuitry, as well as topographic organization of long-range cortical inputs. This scalable approach represents a significant advance in connectomics, enabling circuit reconstruction at unprecedented throughput while maintaining single-cell resolution and molecular identity information.

I presented method is stringently controlled and will be great resource for a very large number of scientists wishing to determine the connectivity of the network they study.

I recommend accepting this beautiful paper as is.

(Remarks on code availability)

Reviewer #2

(Remarks to the Author)
This study by Becalick and Blot applies in situ sequencing to barcoded rabies virus tracing experiments built on the N2c strain. On the one hand, there have now been multiple studies using barcoded rabies virus applied in vivo, in vitro, across strains, using scRNA-seq, and using spatial transcriptomics. Moreover, many preprints are now released that further innovate on these early papers demonstrating barcode nuclear tagging, CRISPR screens, and brainwide mapping. Yet because of the explosion in these variants of the method, I actually view the novelty of this study as contributing to a burgeoning field.

Specifically, the authors have advanced the technology in this work by capturing more neurons in situ than previous studies, and also advancing important principles to detect and quantify neuronal connections locally and at a distance. They also build appropriately on prior studies.

Overall, I view the technical approach and its discussion in the text to be highly detailed, appropriate, and careful. The authors undertook in-depth examinations of their system. They also bring to light new approaches useful to drive forward connectomics at scale including use of the PHP.eB system and high efficiency of starter cell recovery.

Nevertheless, although the paper is well constructed, there are some controls and other analyses that I think would aid in strengthening the paper by having the method inform new biology:

1. Overall, there should be additional visualization and validation of the spatial transcriptomic data, demonstrating where each class of neurons detected sits within the tissue sections, etc. This can be an ED figure.

2. ED5 should be quantified.

3. A few novel candidate connectivity mechanisms or features discovered should be validated on the protein level.

4. What new inferences can be made about the networks detected? Beyond the descriptive analyses in Fig 5 it would help to understand much more deeply the transcriptional changes across the neuronal networks, otherwise the analyses could be done without the spatial transcriptomic component.

5. The authors should show how connectivity changes in response to a perturbation to the visual system.

(Remarks on code availability)

Reviewer #3

(Remarks to the Author)
Novel tools to map connectome between molecularly-defined neurons with high throughput are urgently needed in modern neuroscience. Here in the manuscript titled 'barcoded rabies in situ connectomics for high-throughput reconstruction of neural circuits', Becalick et al reported BRISC, which implemented barcoded rabies viruses to map inputs to hundreds of single neurons in V1 at single cell resolution. While the concept to barcode the connectome with rabies is not novel, it has not been applied to map in vivo connectivity at scale, due to a variety of technical challenges. These challenges include cytotoxicity, limited barcode diversity, and control for starter cell sparsity (sparsity-throughput tradeoff). In this manuscript, Becalick et al developed a novel method to generate high-diversity barcoded rabies libraries that allow for mapping over 1000 starter neurons, and a novel strategy that minimizes connected starter cells while still labeling a reasonable amount of starter cells. Combining optimized barcoding strategies with in situ sequencing, the authors mapped inputs to hundreds of V1 neurons, revealed layer- and cell type-specific wiring patterns, and identified topographic maps in high visual areas. Overall, this research has made significant advances in barcoded rabies techniques, and will provide valuable tools for people interested in neural circuits, neural development, or neuroanatomy. Therefore, it is suitable for publication in Nature Communications. However, there are still several concerns that remain to be addressed.
1. In Line 442, the authors claimed that the majority of presynaptic cells were connected to one starter cell. Does this reflect biological truth or technical bias? When a cell is already infected by barcode from postsynaptic neuron A, is it more difficult to be infected by a second barcode from postsynaptic neuron B? The authors discussed a related issue in line 448, but did not explicitly address this concern. This also raises a more general question: is barcoded rabies suitable for studying synaptic divergence?
2. In Fig. 3F, the authors pointed out that N2cG(H2B-mCherry) expression levels influence tracing efficiencies. Therefore, I assume that some normalization (to mCherry expression) might help when analyzing the data (e.g. most panels in Fig.5). But I guess it also depends on the effect size and variance of mCherry expression.
3. When analyzing topographic maps, the authors mainly focused on the azimuth. How about elevation?
4. The strategies described in the section 'generating high diversity barcoded rabies libraries' are not very friendly to readers without expertise in rabies packaging. I suggest to make some cartoon diagrams to explain the key strategies here.
5. In the section 'controlling starter neuron density', many results and analyses (e.g. Fig.1g-j) are based on the intracerebral strategy. I recommend add corresponding panels for Fig. 1g,h,j for the intravenous strategy. Also, when estimating the proportion of cells present in the sequenced volume, bulk tracing data (intracerebral injection) were used. It is more reasonable to use intravenous injection data.
6. I recommend show some in situ gene expression data in Fig2, and specify how many genes are selected for sequencing in the main text.
7. It might be better to put Fig S1A-B to the main figure.

(Remarks on code availability)

Version 1:

Reviewer comments:

Reviewer #2

(Remarks to the Author)
The authors addressed some of my comments and left protein validation, network validation, and network detection to the future.

(Remarks on code availability)

Reviewer #3

(Remarks to the Author)
The authors have addressed all my concerns in the response letter. I recommend accepting this paper.

(Remarks on code availability)

Open Access This Peer Review File is licensed under a Creative Commons Attribution 4.0 International License, which permits use, sharing, adaptation, distribution and reproduction in any medium or format, as long as you give appropriate credit to the original author(s) and the source, provide a link to the Creative Commons license, and indicate if changes were made.
In cases where reviewers are anonymous, credit should be given to 'Anonymous Referee' and the source.
The images or other third party material in this Peer Review File are included in the article’s Creative Commons license, unless indicated otherwise in a credit line to the material. If material is not included in the article’s Creative Commons license and your intended use is not permitted by statutory regulation or exceeds the permitted use, you will need to obtain permission directly from the copyright holder.
To view a copy of this license, visit https://creativecommons.org/licenses/by/4.0/

Reviewer #1 (Remarks to the Author):

The paper by Becalick et al describes the development of Barcoded Rabies In Situ Connectomics (BRISC), a method for high-throughput mapping of neural connections. The technique uses rabies viruses carrying random molecular barcodes to simultaneously trace the synaptic inputs of hundreds of individual neurons. The authors generated high-diversity barcoded rabies libraries capable of uniquely labeling over 1,000 neurons, controlled starter neuron density through systemic AAV delivery to minimize barcode transmission between starters, and developed an in situ sequencing approach to read out barcodes while preserving spatial information. Applied to mouse primary visual cortex, BRISC mapped inputs onto 385 starter neurons, identifying 7,814 putative synaptic connections. The results revealed layer-specific and cell-type-specific local connectivity patterns consistent with known V1 circuitry, as well as topographic organization of long-range cortical inputs. This scalable approach represents a significant advance in connectomics, enabling circuit reconstruction at unprecedented throughput while maintaining single-cell resolution and molecular identity information.

I presented method is stringently controlled and will be great resource for a very large number of scientists wishing to determine the connectivity of the network they study.

I recommend accepting this beautiful paper as is.

Thank you for your enthusiastic assessment of our work!

Reviewer #2 (Remarks to the Author):

This study by Becalick and Blot applies in situ sequencing to barcoded rabies virus tracing experiments built on the N2c strain. On the one hand, there have now been multiple studies using barcoded rabies virus applied in vivo, in vitro, across strains, using scRNA-seq, and using spatial transcriptomics. Moreover, many preprints are now released that further innovate on these early papers demonstrating barcode nuclear tagging, CRISPR screens, and brainwide mapping. Yet because of the explosion in these variants of the method, I actually view the novelty of this study as contributing to a burgeoning field.

Specifically, the authors have advanced the technology in this work by capturing more neurons in situ than previous studies, and also advancing important principles to detect and quantify neuronal connections locally and at a distance. They also build appropriately on prior studies.

Overall, I view the technical approach and its discussion in the text to be highly detailed, appropriate, and careful. The authors undertook in-depth examinations of their system. They also bring to light new approaches useful to drive forward connectomics at scale including use of the PHP.eB system and high efficiency of starter cell recovery.

We appreciate the reviewer's assessment of the importance of this study within the growing body of work using barcoded rabies viruses and thank them for highlighting key aspects of our work. Using barcoded rabies viruses to study neuronal connectivity depends on unique labelling of starter neurons by different barcode sequences. Our study represents a significant step forward compared to prior work by implementing new strategies for controlling starter cell

density, maintaining high diversity of barcode libraries and measuring the rate of double labelling in each experiment.

> Nevertheless, although the paper is well constructed, there are some controls and other analyses that I think would aid in strengthening the paper by having the method inform new biology:

> 1. Overall, there should be additional visualization and validation of the spatial transcriptomic data, demonstrating where each class of neurons detected sits within the tissue sections, etc. This can be an ED figure.

We have added an additional extended data figure (Extended Data Fig. 6) that visualises the distribution of each class of neurons throughout the sequenced volume of tissue, demonstrating that the identified cell types display the expected laminar biases (lines 333-334). Although the main focus of the manuscript is on the key innovations of BRISC, which enable unique barcode labelling of large numbers of starter cells, we include these data to demonstrate the compatibility of BRISC with readout of the cells' endogenous gene expression patterns.

> 2. ED5 should be quantified.

The quantification of the number of labelled cells was included in the main text (lines 385-390). We have also added this information directly to Extended Data Fig. 5 (now Extended Data Fig. 7), detailing the number of cells detected for each sample.

> 3. A few novel candidate connectivity mechanisms or features discovered should be validated on the protein level.

We appreciate the reviewer's suggestion. The goal of this manuscript is to develop and validate BRISC as a high-throughput approach for mapping monosynaptic inputs to large numbers of individually barcoded starter neurons while preserving spatial information via in situ sequencing. Accordingly, our analyses focus on connectivity patterns (e.g. layer- and cell-type-associated input distributions and the spatial/topographic organization of long-range inputs) derived by matching barcodes between pre- and post-synaptic cells. We do not propose any specific molecular mechanisms underlying synapse formation or specificity.

We acknowledge that differential expression of mRNAs between cell types may not necessarily translate to differences at the protein level. However, such differentially expressed genes still provide a useful signal for cell type classification. We have amended the discussion to clarify this point (lines 709-717).

> 4. What new inferences can be made about the networks detected? Beyond the descriptive analyses in Fig 5 it would help to understand much more deeply the transcriptional changes across the neuronal networks, otherwise the analyses could be done without the spatial transcriptomic component.

The primary aim of this manuscript is to establish BRISC as a scalable approach for mapping monosynaptic inputs to hundreds of individually barcoded starter neurons in situ, and to validate that the resulting connectivity maps recapitulate known wiring principles.

We agree that that deeper analyses of gene expression programmes associated with specific connectivity patterns are an exciting future direction. In this manuscript, we selected a targeted marker panel to classify major cortical cell classes and interneuron subclasses to demonstrate

the ability of BRISC to identify cell-type-specific connectivity patterns (Fig. 5h–j). Systematically relating the expression patterns of single neurons to their input connectivity would require sequencing broader panel of genes. This would be an exciting future application of BRISC, but falls beyond the scope of this manuscript.
We also note that the spatial component provides value beyond what could be obtained from non-spatial sequencing: it preserves the laminar position, local neighbourhood relationships, and topographic organization of long-range inputs (e.g., Figs. 4–6). We clarify this motivation and the intended scope in the text and discuss the investigation of transcriptome-connectivity relationships as a future application of BRISC (lines 706-722).

5. The authors should show how connectivity changes in response to a perturbation to the visual system.

We agree that assessing perturbation-induced changes in connectivity is an important and compelling application of BRISC and appreciate the reviewer's suggestion as it aligns closely with our motivation for developing this method. Its scalability makes comparative connectomics across conditions (e.g., sensory perturbations, learning, development, or disease models) tractable. However, identifying changes in response to a perturbation would be a substantial undertaking – controlling for individual variability requires mapping connections across several mice per experimental group. While this is tractable using BRISC, performing adequate controls and analysing the results of such an experiment would constitute a separate study. This falls beyond the scope of the current manuscript, which is focused on the development and validation of the BRISC methodology itself.

Reviewer #3 (Remarks to the Author):

Novel tools to map connectome between molecularly-defined neurons with high throughput are urgently needed in modern neuroscience. Here in the manuscript titled 'barcoded rabies in situ connectomics for high-throughput reconstruction of neural circuits', Becalick et al reported BRISC, which implemented barcoded rabies viruses to map inputs to hundreds of single neurons in V1 at single cell resolution. While the concept to barcode the connectome with rabies is not novel, it has not been applied to map in vivo connectivity at scale, due to a variety of technical challenges. These challenges include cytotoxicity, limited barcode diversity, and control for starter cell sparsity (sparsity-throughput tradeoff). In this manuscript, Becalick et al developed a novel method to generate high-diversity barcoded rabies libraries that allow for mapping over 1000 starter neurons, and a novel strategy that minimizes connected starter cells while still labeling a reasonable amount of starter cells. Combining optimized barcoding strategies with in situ sequencing, the authors mapped inputs to hundreds of V1 neurons, revealed layer- and cell type-specific wiring patterns, and identified topographic maps in high visual areas. Overall, this research has made significant advances in barcoded rabies techniques and will provide valuable tools for people interested in neural circuits, neural development, or neuroanatomy. Therefore, it is suitable for publication in Nature Communications. However, there are still several concerns that remain to be addressed.

Thank you for your constructive comments.

1. In Line 442, the authors claimed that the majority of presynaptic cells were connected to one starter cell. Does this reflect biological truth or technical bias?

When a cell is already infected by barcode from postsynaptic neuron A, is it more difficult to be infected by a second barcode from postsynaptic neuron B? The authors

discussed a related issue in line 448, but did not explicitly address this concern. This also raises a more general question: is barcoded rabies suitable for studying synaptic divergence?

To ensure unique labelled of starter neurons, we tightly constrain their number. Consequently, only a small fraction of neurons are labelled by transsynaptic spread of the virus. Therefore, we expect most presynaptic cells to only contain one barcode. However, infection by rabies virus may also induce superinfection exclusion, making cells resistant to repeated infection by virions carrying different barcodes. We have now compared the number of presynaptic cells expressing multiple barcodes to that expected from a Poisson model of independent infection (Extended Data Fig. 9). We observed more multiple infected cells than expected from this model, suggesting that, under our conditions, being infected by one barcode does not make presynaptic cells substantially less likely to express a second one. We have amended the manuscript to discuss this explicitly (lines 474-486).

However, detecting synaptic divergence would require labelling starter cells at a density, where a large fraction of potential presynaptic cells are infected through transsynaptic spread. This would likely result in a high rate of transmission of barcodes between starter cells, making barcoded rabies tracing unsuitable for studying synaptic divergence without further technical developments.

2. In Fig. 3F, the authors pointed out that N2cG(H2B-mCherry) expression levels influence tracing efficiencies. Therefore, I assume that some normalization (to mCherry expression) might help when analyzing the data (e.g. most panels in Fig.5). But I guess it also depends on the effect size and variance of mCherry expression.

While the level of mCherry expression correlates with tracing efficiency, a better estimate of tracing efficiency is directly provided by the number of detected presynaptic cells. Therefore, the input fractions in the analyses in Fig. 5 are calculated on a cell-by-cell basis. For example, to calculate the proportion of inputs originating from different cortical layers, we divide the number of inputs from each layer by the total number of presynaptic cells across layers for each starter cell. We then calculate mean input fraction by averaging across starter cells. This takes into account variability in tracing efficiency across starter cells and ensures that individual starter cells with high tracing efficiency do not disproportionately skew the calculated input fractions.

3. When analyzing topographic maps, the authors mainly focused on the azimuth. How about elevation?

The orientation of the tissue volume that was sequenced in these experiments spans a large region in the mediolateral axis, but a comparably small region in the anteroposterior axis of the primary visual cortex. This means that the section of the topographic map that was captured in this dataset spans a large extent of the azimuth plane, but a small part of the plane of elevation. For this reason we initially focused on mediolateral location and azimuth. We have included a corresponding plot of the connectivity corresponding to elevation and anterior-posterior position here.

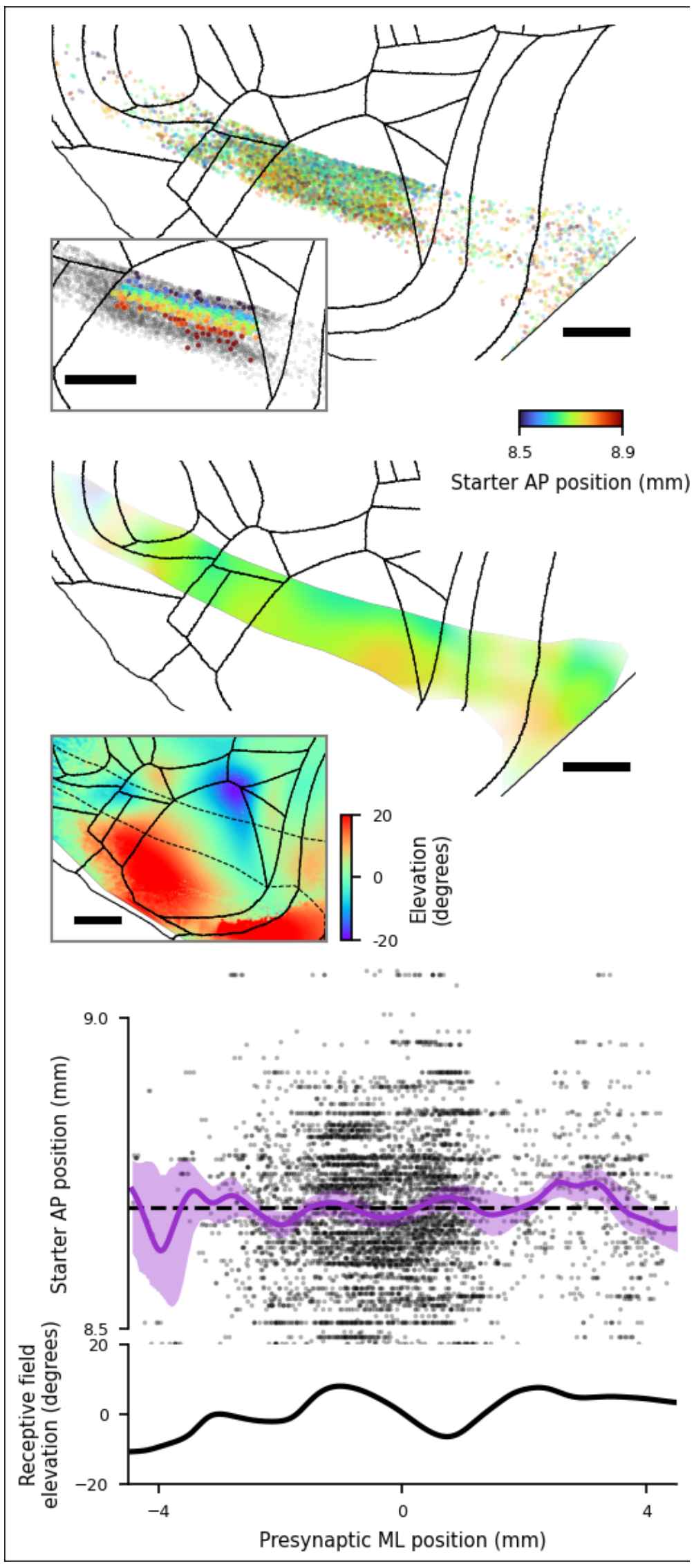

4. The strategies described in the section ‘generating high diversity barcoded rabies libraries’ are not very friendly to readers without expertise in rabies packaging. I suggest to make some cartoon diagrams to explain the key strategies here.

We have added a diagram as suggested (Fig. 1c) and expanded the corresponding text (lines 131-167) to clarify the viral production protocol to readers without expertise in the field.

5. In the section ‘controlling starter neuron density’, many results and analyses (e.g. Fig.1g-j) are based on the intracerebral strategy. I recommend add corresponding panels for Fig. 1g,h,j for the intravenous strategy. Also, when estimating the proportion of cells present in the sequenced volume, bulk tracing data (intracerebral injection) were used. It is more reasonable to use intravenous injection data.

We have added the corresponding panels for Fig. 1g-j with intravenous delivery of Cre (Extended Data 2a-c). The number of presynaptic cells per starters was marginally higher than that measured with the intracortical injection (88.0 presynaptic neurons per starter for intravenous vs 66.6 for intracortical), with a slightly higher proportion of local presynaptic cells (40% for intravenous vs 31%). This result is consistent with the broader and sparser distribution of starter cells achieved with intravenous injections (Van-Tran Minh et al. 2023). As the dense distribution of starters obtained with the intracortical injection ensures a higher

coverage of cell types in the injection site and provides more conservative estimates, these were kept for estimating the proportion of cells present in the sequenced volume.

6. I recommend show some in situ gene expression data in Fig. 2, and specify how many genes are selected for sequencing in the main text.

We have added examples of in situ gene expression data for several canonical marker genes (Fig. 2j) and have amended the main text (line 322) to explain the number of genes selected for sequencing (98 genes).

7. It might be better to put Fig S1A-B to the main figure.

Thank you for the suggestions, we have moved panels Fig S1A-B to Figure 1f,g and updated it to include an additional barcoded rabies virus library from Tan et al., 2025.
